# State Medical Society of Kentucky

**Published:** 1841-10

**Authors:** 


### STATE MEDICAL SOCIETY OF KENTUCKY.

We take the liberty of calling the attention of our readers in this state, to the first annual meeting of this society in January next. Let it not only be well attended, but prolific in good fruits. Reports, full and scientific, should be made on all our summer and autumnal diseases, by observing in different parts of the state; the reading and discussion of which could not fail to be highly instructive. Such a meeting at the commencement would give an impetus to the society, that would carry it on to a higher respectability, than many similar societies have attained. Every county of the state, will, we trust, be represented in the meeting. All should come who are able to leave home—those who can teach, prepared to do so; those who cannot, prepared to listen and be taught. But we should not encourage *elementary* instruction or *popular* harangues. The study of our endemic diseases, in their causes, peculiarities and treatment, is the legitimate object of the society; and should be steadily kept in view. Those who intend to read papers, ought not to defer the preparation of them to the last hour; but, by engaging in it at an early period, give to them the accuracy, fullness and finish, which are equally due to the society, the profession, and themselves. No one should preface his paper with an apology for any other imperfection, than the want of unattainable facts. D.

